# Phase 1 study to access safety, tolerability, pharmacokinetics, and pharmacodynamics of kynurenine in healthy volunteers

**DOI:** 10.1002/prp2.741

**Published:** 2021-03-07

**Authors:** Mohammad Al‐Mahdi Al‐Karagholi, Jakob Møller Hansen, Dalia Abou‐Kassem, Anna Koldbro Hansted, Kumari Ubhayasekera, Jonas Bergquist, László Vécsei, Inger Jansen‐Olesen, Messoud Ashina

**Affiliations:** ^1^ Danish Headache Center Department of Neurology University of Copenhagen Denmark; ^2^ Danish Headache Knowledge Center Rigshospitalet – Glostrup Glostrup Denmark; ^3^ Danish Headache Center Glostrup Research Institute Rigshospitalet Glostrup Faculty of Health and Medical Sciences University of Copenhagen Denmark; ^4^ Analytical Chemistry and Neurochemistry Department of Chemistry ‐ BMC Uppsala University Sweden; ^5^ Department of Neurology and MTA‐SZTE Neuroscience Research Group University of Szeged Szeged Hungary

**Keywords:** epilepsy, glutamat, kynurenic acid, migraine, stroke

## Abstract

The kynurenine pathway (KP) is the main path for tryptophan metabolism, and it represents a multitude of potential sites for drug discovery in neuroscience, including pain, stroke, and epilepsy. L‐kynurenine (LKYN), the first active metabolite in the pathway, emerges to be a prodrug targeting glutamate receptors. The safety, tolerability, pharmacokinetics, and pharmacodynamics of LKYN in humans have not been previously investigated. In an open‐label, single ascending dose study, six participants received an intravenous infusion of 50, 100, and 150 µg/kg LKYN and new six participants received an intravenous infusion of 0.3, 0.5, 1, and 5 mg/kg LKYN. To compare the pharmacological effects between species, we investigated in vivo the vascular effects of LKYN in rats. In humans, LKYN was safe and well‐tolerated at all dose levels examined. After infusion, LKYN plasma concentration increased significantly over time 3.23 ± 1.12 µg/mL (after 50 µg/kg), 4.04 ± 1.1 µg/mL (after 100 µg/kg), and 5.25 ± 1.01 µg/mL (after 150 µg/kg) (*p* ≤ 0.001). We observed no vascular changes after infusion compared with baseline. In rats, LKYN had no effect on HR and MAP and caused no dilation of dural and pial arteries. This first‐in‐human study of LKYN showed that LKYN was safe and well‐tolerated after intravenous infusion up to 5 mg/kg over 20 minutes. The lack of change in LKYN metabolites in plasma suggests a relatively slow metabolism of LKYN and no or little feed‐back effect of LKYN on its synthesis. The therapeutic potential of LKYN in stroke and epilepsy should be explored in future studies in humans.

## Graphical abstract

Study design and outcomes

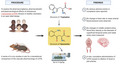

## 1 INTRODUCTION

**FIGURE 1:**
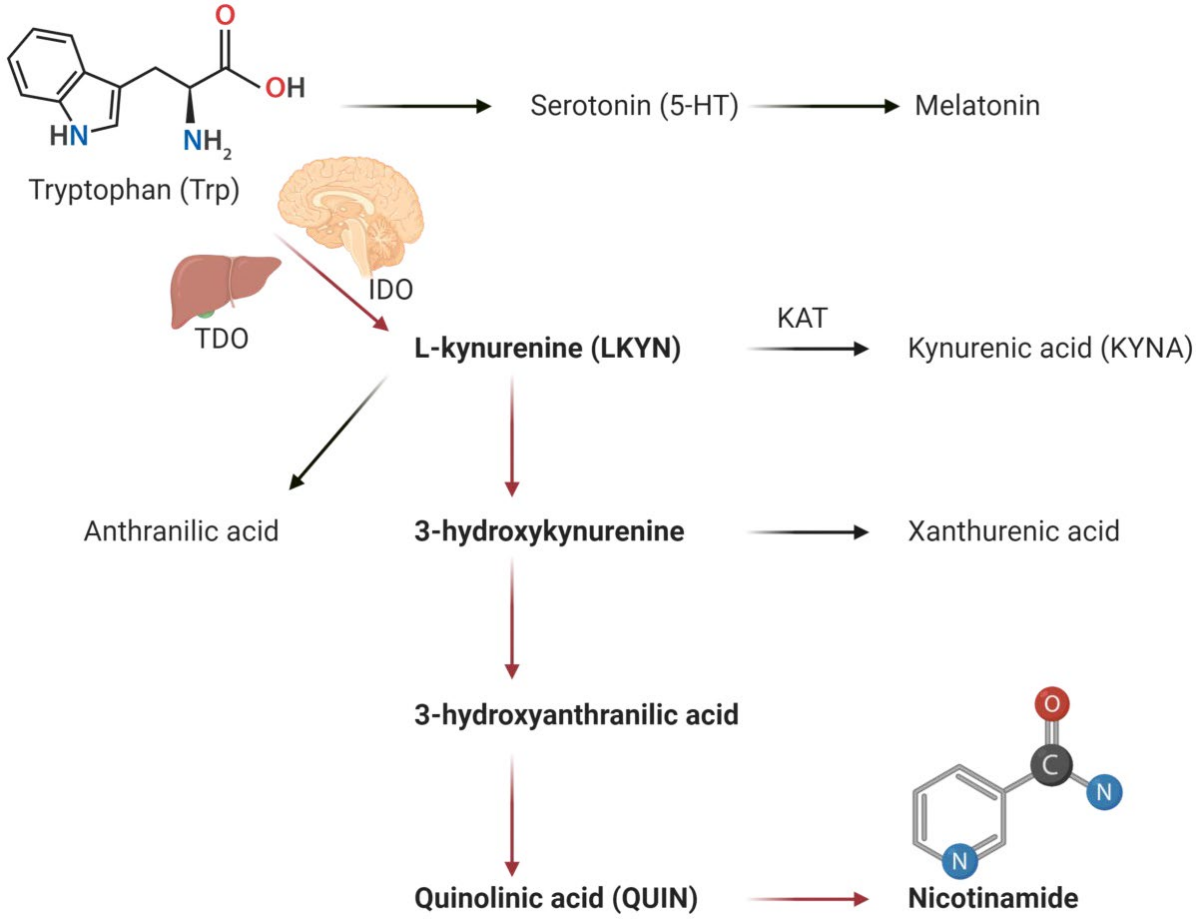
A summary of tryptophan (Trp) metabolism. The kynurenine pathway represents 99% of Trp metabolism. The principal branch (red arrows) for tryptophan metabolism generates L‐kynurenine (LKYN), quinolinic acid (QUIN), and nicotinamide

The kynurenine pathway (KP) is the main route for nonprotein metabolism of the essential amino acid tryptophan (Trp).^1^ The dietary tryptophan intake is transformed biochemically into other compounds such as serotonin and melatonin,^1^ and ~99% is metabolized by the KP.^2^ Oxidative catabolism of Trp in the KP can be initiated by one of three different enzymes that open the indole ring, causing the irreversible loss of Trp^3^; tryptophan‐2,3‐dioxygenase (TDO) in the liver, as well as indoleamine‐2,3‐dioxygenase 1 (IDO1) and 2 (IDO2) extrahepatic.^4^ The principal branch for tryptophan metabolism generates L‐kynurenine (LKYN), quinolinic acid (QUIN), and nicotinamide, whereas, the side branches generate kynurenic acid (KYNA) and xanthurenic acid (Figure 1).^5^ The KP gained a considerable scientific interest by discovering that QUIN could excite neurons in the central nervous system (CNS) by acting as an agonist at the N‐methyl‐D‐aspartate (NMDA) receptor,^6^ while KYNA is the only endogenous NMDA receptor antagonist.^7^ This observation raised the possibility that the KP could be involved in various CNS phenomena, including synaptic plasticity, neurodegeneration,^8^ epilepsy,^9^ and schizophrenia.^10^ Since then, the KP has been implicated in various conditions including cognitive ability,^11^, ^12^ migraine,^13^ inflammation‐associated depressive symptoms,^14^ heart failure,^15^ stroke,^16^ and invasive cancer cells.^17^

LKYN is the source for all the other kynurenine metabolites, and it is readily transported across the blood–brain barrier (BBB) by a neutral amino acid carrier.^18^ KYNA emerges to be a promising therapeutic drug for several neurological disorders,^1^ but its use as a neuroprotective agent is rather restricted due to limited ability to cross the BBB.^18^ Interestingly, the generation of KYNA endogenously in brain slices by perfusion with LKYN has a greater inhibitory action than a similar concentration applied exogenously.^9^ Furthermore, peripheral treatment with LKYN dose‐dependently increases the concentration of KYNA in the brain, offering an opportunity for the treatment of stroke and neurodegenerative disorders.^16^, ^19^, ^20^, ^21^, ^22^, ^23^

However, the safety, tolerability, and physiological effect of LKYN in vivo in human is yet to be elucidated. In the present study, we investigated the pharmacovigilance, pharmacokinetic and pharmacological effects of intravenous LKYN infusion in healthy volunteers. We added a series of *in vivo* studies in rats for a translational comparison of the vascular pharmacology of LKYN.

## 2 MATERIALS AND METHODS

### 2.1 Animal

Experiments using the closed cranial window model were performed on seven male Sprague‐Dawley rats (295–340 g; Taconic, Denmark) under approval number 2014‐15‐0201‐00256 from the Danish Animal Experiments Inspectorate. All rats were group housed in Tecniplast 1354G Eurostandard type IV polycarbonate cages (L*W*H: 60*38*20 cm; Brogaarden, Denmark) using a 12‐hour light/dark cycle with lights on at 06.00 am. Individual opaque red polycarbonate shelters (20*11.5*16 and 15*9*9 cm, respectively), together with an aspen biting stick (10*2*2 cm; Tapvei, Estonia) and piece of hemp rope suspended from the cage lid, were provided in each homecage for retreat and enrichment purposes. Bedding consisted of Enviro‐Dri nesting material (Brogaarden, Denmark). Standard rat chow (Altromin) and tap water were available ad libitum in the animals’ homecage environment. Humidity ranged from 45–65%. All in vivo experiments were performed between 8 am and 4 pm. One unit represents one animal.

#### 2.1.1 Closed cranial window model

The rats were anaesthetized by an intraperitoneal injection of pentobarbital (65 mg/kg). A rectal thermometer connected to a heating pad was used to maintain the body temperature at 37.5°C. The trachea was cannulated and connected to a ventilator (SAR‐830/P Ventilator, CWE Inc.). The femoral vein and artery were cannulated on both sides using BTPE‐10, Polyethylene tubing, 0.011 × 0.024 in (0.28 × 0.60 mm) and BTPU‐040, Polyurethane tubing, 0.025 × 0.040 in (0.63 × 1.02 mm), and secured with suture. The arteries were used for continuous blood pressure measurements and sampling of arterial blood for blood gas analysis. The veins were used for a continuous infusion of anesthesia (pentobarbital 50 mg/ml; 0.15–0.23 ml/hour). A free‐floating carotid catheter for drug infusion was placed in the right carotid artery using tissue glue as previously described.^24^ The rat was placed in a stereotaxic frame, and the right parietal bone was exposed and thinned to transparency using a dental drill. To avoid overheating of the bone and underlying tissues while drilling, the surface of the bone was repeatedly washed with cold saline. The cranial window was then covered with mineral oil to prevent it from drying, and dural and pial arteries were viewed with an intravital microscope consisting of a Kappa CF8/5 digital camera (Kappa optronics GmbH, Gleichen, Germany) connected to a Leica Model MZ 16 microscope with a 0.5X 10445929 Video Objective (Leica Microsystems, Brønshøj, Denmark). The diameter of the arteries was monitored with a video dimension analyzer (V94, Living Systems Instrumentation Inc., Burlington, VT, USA) and recorded and analyzed together with the mean arterial blood pressure with Perisoft (Version 2.5.5; Perimed AB, Järfälla, Sweden). Before initiation of the experiments, the tracheal cannula was connected to a ventilator, a blood gas analysis was made, and the ventilator was adjusted if needed. Another blood gas analysis was made halfway through the experiment.

#### 2.1.2 Experimental protocol

**FIGURE 2:**
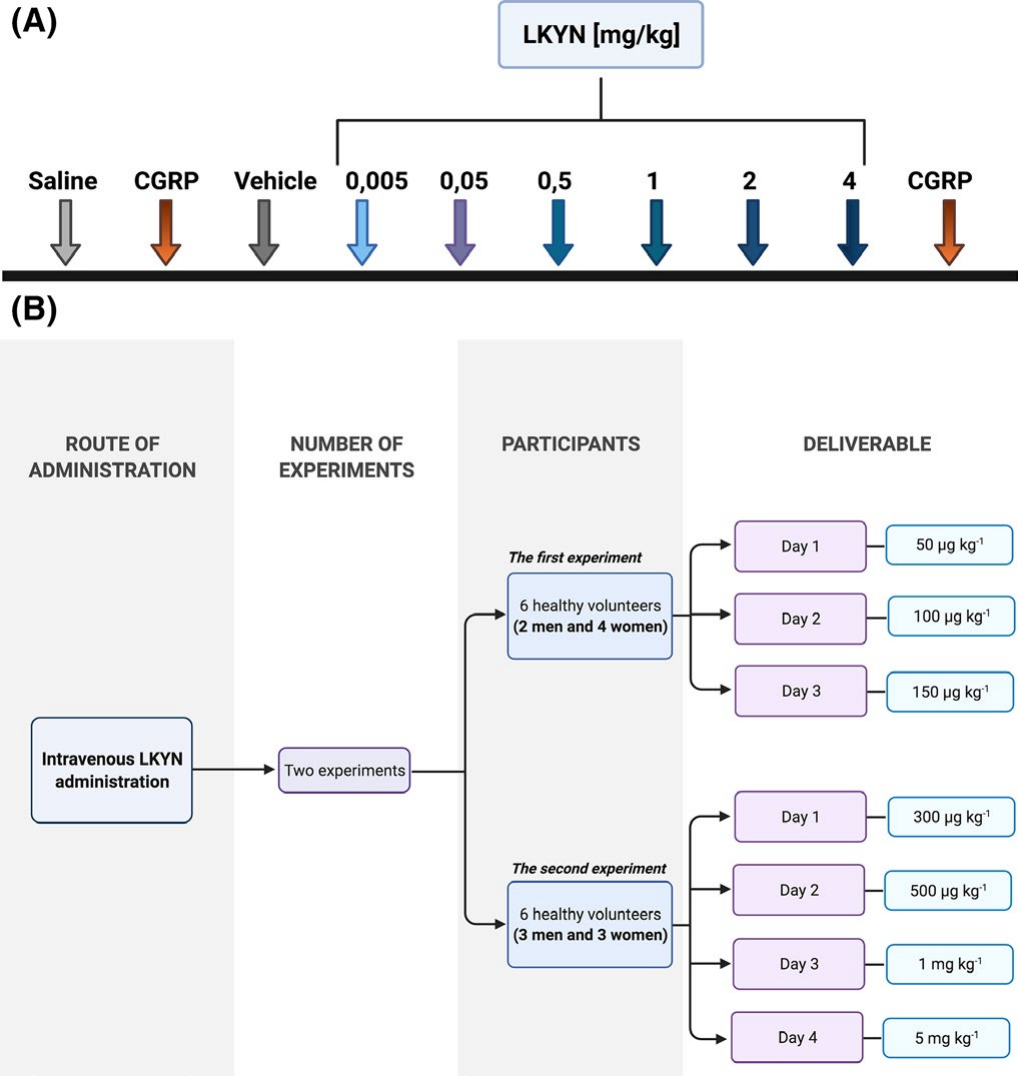
A. Design of the animal experiments. Intracarotid (ic) infusion of saline, CGRP, and vehicle (LKYN) were performed on seven male Sprague‐Dawley rats. There was 10 min between each dose of LKYN. A closed cranial window model was used to measure vascular changes in dural and pial arteries. B. Design of human study. Twelve healthy volunteers; six to the first experiment study and six to the second experiment study. In the first experiment study, the participants received a continuous intravenous infusion of 50, 100, and 150 µg/kg LKYN in the mentioned order over 20 min on three days separated by at least 1 week. In the second experiment study, the participants received a continuous intravenous infusion of 0.3, 0.5, 1, and 5 mg/kg LKYN in the mentioned order over 20 min on four days separated by at least 1 week. Blood and urine samples were collected only during the first experiment study

All infusions were made with a rate of 125 µl/kg/min and a volume of 125 µl/kg. As a standard protocol, the experiments started with an intracarotid (ic) infusion of 250 µl saline followed by CGRP (500 ng/kg ic) as a positive control. The seven male rats then received increasing doses (5 µg/kg, 50 µg/kg, 500 µg/kg, 1 mg/kg, 2 mg/kg, and 4 mg/kg ic) of LKYN (β‐anthraniloyl‐L‐alanine, L‐2‐Amino‐4‐(2‐aminophenyl)‐4‐oxobutanoic acid, Sigma‐Aldrich, Denmark). There was 10 min between each dose of LKYN. After the last dose of LKYN, the viability of the system was again tested by the administration of a second CGRP infusion of the same dose as described above. Thereafter, while still in anesthesia the rats were sacrificed by infusion of 1 M KCl until visible cardiac arrest was obtained (Figure 2A).

#### 2.1.3 Drugs

CGRP (Tocris Bioscience, Bio‐Techne Ltd, UK) was dissolved in saline to a concentration of 0.5 mg/ml. LKYN (Sigma‐Aldrich, Denmark) was dissolved in 0.1 M NaOH. CGRP and LKYN were further diluted with saline or 0.1 M NaOH to their final concentration just prior to the experiment.

#### 2.1.4 Data treatment and statistical analysis

The effectiveness of the test substances is based on measurements of three parameters: changes in the diameter of dural and pial arteries and changes in mean arterial blood pressure. The artery diameter was measured in arbitrary units and mean arterial blood pressure in mmHg. Dilation of the arteries and changes in mean arterial blood pressure were calculated as percentage change from the baseline, which is defined as the average of the 60 seconds preceding administration of test substance. Vessel diameter was measured at the peak response occurring 1 to 2 minutes after drug administration. The group size was estimated as a function of the desired effect size (approximately 50% change versus corresponding vehicle treatment) where we assumed a significance level of 5% and a power of 90%.^25^

Statistical analyses were made using GraphPad Prism 8 with the mixed effects model for datasets with missing values (a missing value in the second CGRP administration group) were used to analyze the overall effects of treatments followed by Bonferroni's multiple comparisons test. Groups were considered significantly different when the p‐value was less than 0.05. All values are given as mean ± SEM.

### 2.2 Human

We recruited a total of twelve healthy volunteers, six to the first experiment and six to the second experiment. None of the participants had previously participated in similar provocation studies. All participants were recruited through the Danish test subject Web site (www.forsogsperson.dk). All participants gave written informed consent before inclusion. The female patients were required to have sufficient contraception (contraceptive pill or intrauterine device/system (IUD/IUS)). Exclusion criteria were any type of primary headache (except episodic tension‐type headache no more than one day per month), previous serious somatic or psychiatric diseases, or intake of daily medication including prophylactic migraine treatment, except oral contraceptives. A full medical examination and ECG were performed on the day of recruitment.

The study was approved by Capital Region Ethics Committee of Denmark (H‐16033148) and the Danish Data Protection Agency, and was conducted according to the Declaration of Helsinki of 1964, as revised in 2008. The study was also registered at ClinicalTrials.gov (NCT03212430).

#### 2.2.1 Data availability

The data that support the findings of this study are available from the corresponding author, upon reasonable request.

#### 2.2.2 Experimental design

##### Experiment 1

In an open‐label design, the participants received a continuous intravenous infusion of 50, 100, and 150 µg/kg LKYN (manufactured by Sigma‐Aldrich under conditions and practices required by the good manufacturing practice (GMP) regulations, reference number CSQ‐23526R2) over 20 minutes (min) on three days separated by at least 1 week (Figure 2B). LKYN was dissolved in 0.1 M NaOH and was further diluted with saline to its final concentration just prior to the experiment. The final pH was around 7.4.

##### Experiment 2

In an open‐label design, the participants received a continuous intravenous infusion of 0.3, 0.5, 1, and 5 mg/kg LKYN (manufactured by Sigma‐Aldrich under conditions and practices required by the good manufacturing practice regulations (GMP), reference number CSQ‐23526R2) over 20 minutes on four days separated by at least 1 week (Figure 2B). LKYN was dissolved in 0.1 M NaOH and was further diluted with saline to its final concentration just prior to the experiment. The final pH was around 7.4.

All participants arrived nonfasting at the clinic between 8:00 a.m. and 08:30 a.m. The experiment was postponed, if the patients had any type of headache 48 hours before the start of the study. The participants were placed in the supine position, and a venous catheter was inserted into the left and right antecubital vein for drug infusion and blood samples.

The participants then rested for at least 30 minutes before baseline measurements of blood pressure (ProPac Encore; Welch Allyn Protocol), heart rate (HR), and ECG (Cardiofax V; Nihon‐Kohden, Shinjuku‐ku, Tokyo, Japan) were performed, and the infusion of 20 ml of LKYN started using a time and volume‐controlled infusion pump.

Middle cerebral artery blood flow velocity (V_MCA_), left superficial temporal artery (STA) diameter, left radial artery (RA) diameter, and end‐tidal partial pressure of CO_2_ (PetCO_2_) were recorded before, 10 minutes after, and then every 20 minutes until 120 min following the beginning of infusions. Mean V_MCA_ was recorded bilaterally using transcranial Doppler (TCD; Doppler BoxX, DWL, Singen, Germany) with hand‐held 2‐MHz probes as previously described.^26^, ^27^ Diameter of the frontal branch of the superficial temporal artery (STA) and the radial artery (RA) was measured by a high‐resolution ultrasonography unit (Dermascan C; Cortex Technology, Hadsund, Denmark: 20 MHz, bandwidth 5 MHz) as previously described.^28^, ^29^ End‐tidal CO_2_ (P_et_CO_2_ was recorded simultaneously with TCD recordings using an open mask that caused no respiratory resistance (ProPaq Encore; Welch Allyn Protocol, Beaverton, OR, USA.

Headache characteristics, including intensity and accompanying symptoms, vital signs, and adverse events were recorded before and then every 10 minutes until 120 minutes after the beginning of infusion. Headache intensity was recorded on numerical rating scale (NRS) from 0 to 10. The participants were discharged from the hospital after finishing the measurements and asked to complete a headache diary every hour until 24 hours after start of infusion. The diary included headache characteristics and accompanying symptoms, any rescue medication, adverse events, and premonitory symptoms (unusual fatigue, yawning, neck stiffness, mood swings).

#### 2.2.3 Measurement of LKYN and related metabolites

During the first experiment, blood and urine samples were collected for LKYN measurement. Due to limited resources, we only assessed LKYN pharmacokinetic properties during experiment 1. Blood samples (10 ml) were collected at baseline and 10, 20, 40, 60, 80, 100, and 120 min after start of infusion in ice‐chilled K3‐EDTA tubes (Greiner Bio‐One International GmbH). Plasma was separated by centrifugation (2000 *g* in 10 minutes) at 4°C, aliquoted, and immediately frozen at −80°C. Urine samples (10 ml) for LKYN measurement were collected at baseline, 120 min and 24 hours. They were centrifugated (2000 *g* in 10 minutes) at 4°C, aliquoted, and immediately frozen at −80°C.

Kynurenine, kynurenic acid, and tryptophan were analyzed using Ultra‐Performance Liquid Chromatography (Waters ACQUITY® UPLC, Milford, MA) coupled with QExactive Orbitrap mass spectrometer (Thermo Scientific, Bremen, Germany). A slightly modified method of liquid–liquid extraction was used.^30^ In brief, a 100 µl of plasma or 200 µl of urine was spiked with 100 µl mixture of a corresponding deuterated internal standard mixture (20 µg/ml). The samples are deproteinized by the addition of 200 µl and 400 µl of ice‐cooled acetonitrile with 1% formic acid, respectively, then vortexed for 1 minutes. Sample is incubated for 20 min at −80°C to ensure a complete protein precipitation, followed by a centrifugation at 13,000 rpm for 10 min at 4°C. The supernatant was transferred to a clean tube and evaporated under stream of nitrogen, and the sample was reconstituted into mobile phase B.

Separation of these analytes was accomplished using the BEH C18 (2.1 × 100 mm, 1.7 µm) column at 40°C. A binary gradient consisting of solvent A (0.1% formic acid in methanol) and solvent B (methanol) was applied. Samples were separated in gradient elution. After 2 minutes of isocratic elution with 98% A, a 2‐ to 8‐min gradient elution followed, ending at 100% B. At the end of the elution, the column was cleaned with 100% methanol for 1 minute.

Mass spectrometric detection was performed using electrospray ionization in the positive mode. The heated electrospray ionization source (HESI) was operated at a voltage of 3.5 kV and temperature set at 325°C, with the sheath gas and the auxiliary gas flow rate of 40 and 5 arbitrary units, respectively. Acquisition was performed a mass range of 50–800 m/z with 30,000 mass resolution. The quantification of the analytes was based on a multiple reaction monitoring (MRM) coupled to stable isotope dilution. The limit of quantification (LOQ) and coefficient of variation of kynurenine, kynurenic acid and tryptophan were 0.5, 0.1, and 0.1 ng/mL and 5.4%, 2.2%, and 3.4%, respectively. The recovery of kynurenine, kynurenic acid, and tryptophan was 87%, 95%, and 90%, respectively. Linear rage of quantification for kynurenine, kynurenic acid, and tryptophan was 0.05–6500, 50–10000, and 5–15000 ng/mL, respectively. All data were acquired, analyzed, and processed using the Thermo Scientific Xcalibur software (version 4.1, Thermo Scientific, Bremen, Germany).

#### 2.2.4 Data analysis and statistics

All values are presented as mean values ±SD, except headache scores which are presented as median values. Baseline was defined as T_0_ before the start of infusion of each dose. We calculated AUC according to the trapezium rule^31^ to obtain a summary. Difference in AUC for headache intensity scores, HR and mean arterial pressure (MAP), V_MCA_ (0–2 hours), STA diameter and RA diameter between the seven doses was tested using the Friedman test. All analyses were performed with SPSS Statistics version 19 for Windows, and P‐value <0.05 was considered as the level of significance.

## 3 RESULTS

### 3.1 Rats

#### 3.1.1 Dural arteries

**FIGURE 3:**
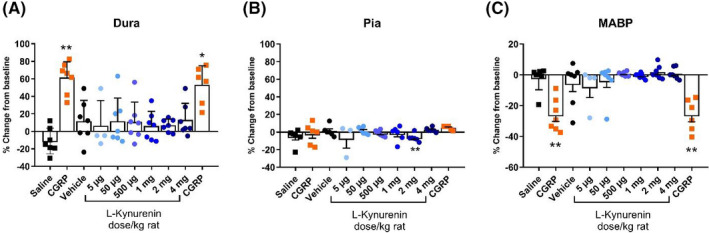
Changes in dural and pial artery diameter and mean arterial blood pressure after infusion of LKYN. Change in diameter of dural arteries (A), pial arteries (B), and mean arterial blood pressure (MABP) (C) after infusion of CGRP (orange squares) and increasing doses (5 µg/kg to 4 mg/kg) of LKYN (blue bullets; the darker the blue color, the higher is the dose) as compared to saline and vehicle infusion. Statistical analysis with mixed‐effect analysist *p < 0.05; **p < 0.01 as compared to saline (CGRP) and vehicle (LKYN). The number of experiments is 6–7 for CGRP and 4–7 for LKYN

CGRP infusion to anesthetized rats caused a significant increase in dural artery diameter of 61.4 ± 6.8% as compared to −11.1 ± 5.5% (n = 7) after saline infusion (*p *<* *0.01) (Figure 3A). There was no difference (*p = *0.56; n = 7) between the first and second (53.1 ± 8.9%) administration of CGRP. Cumulative administration of LKYN caused no dilation of dural arteries as compared to the response induced by its vehicle (11.5 ± 9.0%) (*p > 0*.*99*; n = 4–7). The measured values after LKYN administration varied between 6.0 ± 6.4% at 1 mg/kg and 13.0 ± 7.2% at 4 mg/kg (Figure 3A).

#### 3.1.2 Pial arteries

Intra carotid artery infusion of CGRP caused no increase in pial artery diameter. The effect after the first CGRP infusion was −2.2 ± 4.8% (n = 6) as compared to saline that induced a response of −5.3 ± 3.6% (n = 6) (Figure 3B). There was no difference between the two administrations of CGRP (*p = *0.69; n = 5–6) with the second CGRP response amounting to 4.7 ± 1.1% (n = 5). As found in dural arteries, there was no dilation after infusion of LKYN in cumulative doses (*p > *0.05). On the contrary, a significant (*p *<* *0.01) constriction of −6.5 ± 1.8% (n = 6) was observed at 2 mg/kg LKYN when compared to the vehicle response of 1.1 ± 2.5% (n = 6). We believe this is a false‐positive outcome as the change in diameter was very small and all other doses of LKYN were highly nonsignificant (*p > *0.999) when compared to the vehicle response (Figure 3B).

#### 3.1.3 Mean arterial blood pressure

When compared to the vehicle response of −2.0 ± 2.9%, the first and second infusion of CGRP significantly (*p *<* *0.01) decreased mean arterial blood pressure (MABP) to −26.2 ± 4.0% (n = 7) and −26.2 ± 4.3% (n = 6), respectively (Figure 3C). Cumulative administration of LKYN in doses ranging from 5 µg/kg to 4 mg/kg had no significant effect (*p > *0.999 on all comparisons to vehicle, n = 4–7) with responses between 1.8 ± 1.6% at 2 mg/kg and −8.0 ± 6.7% mg/kg at 5 µg/kg (Figure 3C).

### 3.2 Humans

Six healthy volunteers (4 women and 2 men) completed the first experiment (Figure 2B). The mean age was 21 years (range 20–22) and mean weight 69 kg (range 58–76). Six healthy volunteers (3 women and 3 men) completed the second experiment (Figure 2B). The mean age was 23 years (range 22–25) and mean weight 74.5 kg (range 52–93). There was no carry‐over or period effect for baseline values of headache, HR, MAP, V_MCA_, STA, or RA.

#### 3.2.1 Safety and tolerability

**FIGURE 4:**
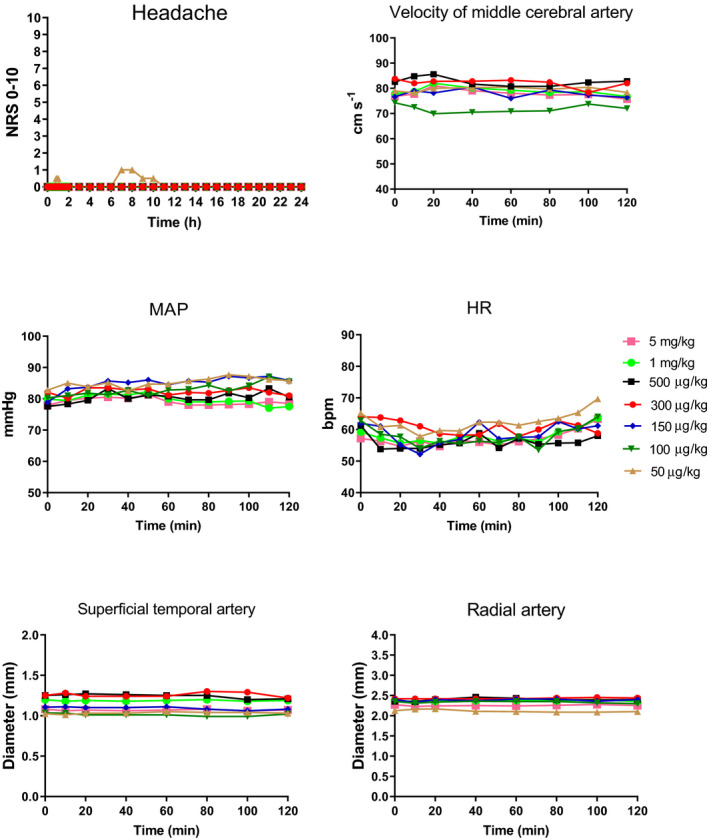
Headache and vascular changes after the infusion of LKYN in humans. Median headache intensity measured with numerical rating scale ((NRS), NRS; 0, no headache; 1, a feeling of pressure; 10, worst imaginable headache). Only mild headache (NRS median =1) was provoked after the first dose. The median headache after the following doses was =0. No significant change (*p > *0.05) was observed in mean changes in middle cerebral artery blood flow velocity (V_MCA_), mean heart rate (HR), mean blood pressure (MAP) superficial temporal artery (STA), and radial artery (RA)

The participants tolerated the used doses well and no serious adverse events or complaints were reported. Five of 6 participants (83%) developed mild headache (NRS <4) after the first dose 50 µg/kg. Two of 6 participants (33%) developed mild headache (NRS <4) after 100 µg/kg and 150 µg/kg. One of 6 (17%) developed mild headache (NRS <4) after 300 µg/kg and 500 µg/kg. None of the participants developed headache after 1 mg/kg or after 5 mg/kg (Figure 4). Other adverse events reported were unusual fatigue and sedation (3 after 150 µg/kg and 300 µg/kg dose, 4 after 500 µg/kg, 1 mg/kg, and 5 mg/kg dose).

#### 3.2.2 Pharmacokinetic effects

**FIGURE 5:**
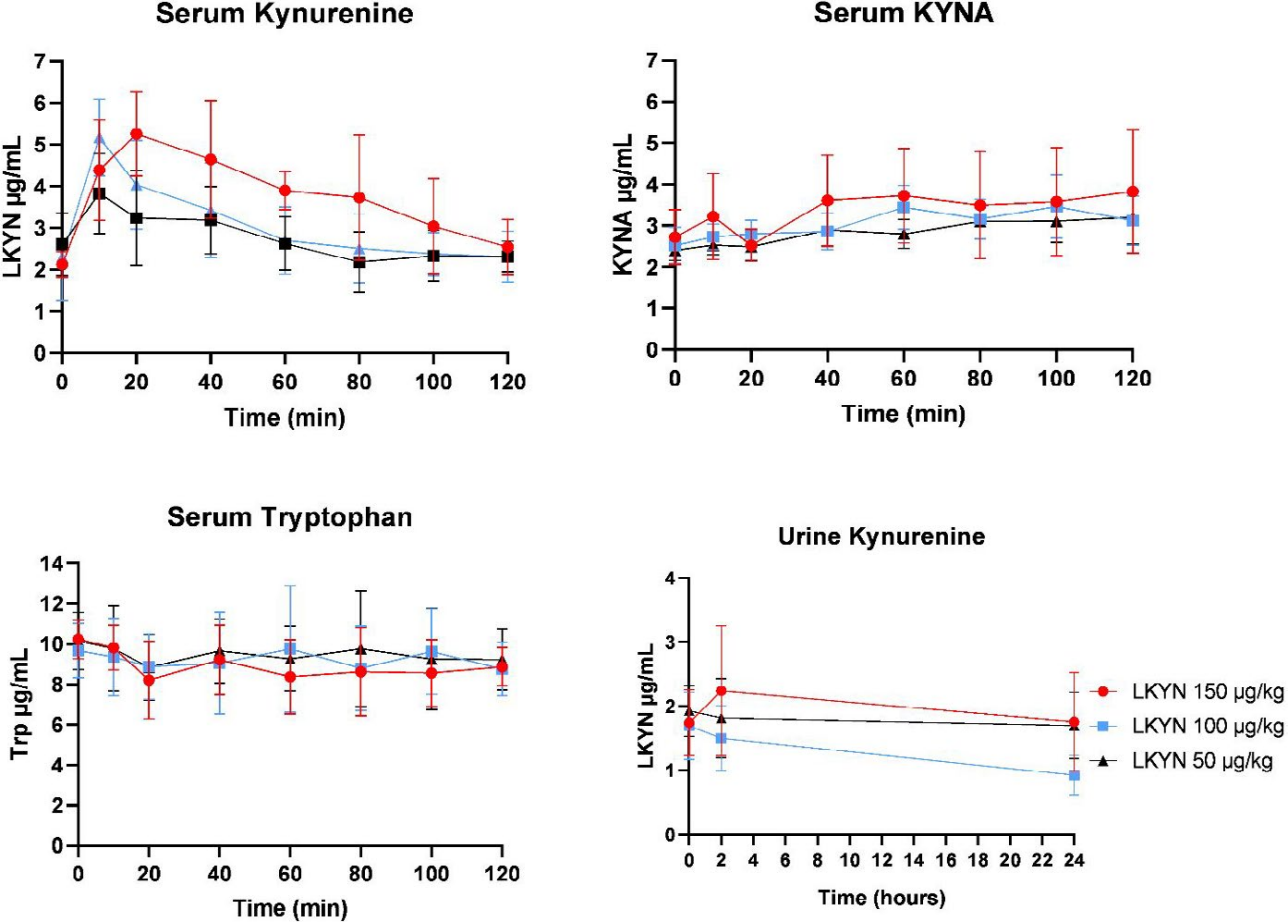
Serum and urine concentrations after the first experiment of human study. LKYN plasma concentration after infusion of LKYN over time for 150 µg/kg (*p* ˂ 0.0001), 100 µg/kg (*p* ˂ 0.0001), and 50 µg/kg (*p = *0.001). Whereas no change was observed in plasma concentrations of KYNA or tryptophan (*p* > 0.05), tryptophan and KYNA plasma concentration and LKYN urine concentration remain largely unchanged. All values are given as mean ± SEM

LKYN plasma concentration increased significantly over time after infusion 3.23 ± 1.0 µg/ml (after 50 µg/kg (*p* = 0.001)), 4.04 ± 1.1 µg/mL (after 100 µg/kg (*p* ˂ 0.0001)), and 5.25 ± 1.01 µg/mL (after 150 µg/kg (*p* ˂ 0.0001)). Immediately after the 20 min, intravenous infusion of 150 µg/kg LKYN plasma levels peaked at 5.25 ± 1.01 µg/mL compared with 2.19 ± 0.31 µg/ml at baseline. Thereafter, LKYN plasma concentration decreased exponentially. The mean elimination constant (K_e_) (calculated using the equation: ln(C) = ln(C_0_) ‐ K_e_ x t) was 0.0074 ± 0.004 min^−1^, and thus, half‐life was 94 minutes (Table 1). While there was a trend for KYNA plasma concentration and LKYN urine concentration to be increased after LKYN infusion, they did not reach a statistical difference (*p > *0.05), and tryptophan plasma concentration remained stable (*p > *0.05) (Figure 5). ANOVA showed significant changes in LKYN plasma concentration after infusion of LKYN over time for 150 µg/kg (*p* ˂ 0.0001), 100 µg/kg (*p* ˂ 0.0001), and 50 µg/kg (*p = *0.001).

**TABLE 1:** Pharmacokinetic of intravenous infusion of LKYN (150 µg/kg) in the first experiment in humans

Participant	C_max_ (µg/mL)	K_e_ (min^−1^)	AUC ((µg/mL) x min)	AUC without baseline
1	3.94	0.002523	442	204.7
2	4.67	0.01216	367.1	124.3
3	5.55	0.005643	573.1	308.9
4	6.95	0.009071	594.4	316.6
5	5.08	0.005485	457.8	211.8
6	5.34	0.009561	353.2	118.5
Mean ± SD	5.25 ± 1.01	0.0074 ± 0.004	464.6 ± 101.1	214.1 ± 85.8

#### 3.2.3 Hemodynamic variables

We found no change in HR, mean arterial blood pressure (MAP), V_MCA_, and STA and RA diameter after LKYN infusion compared with baseline (*p > *0.05) (Figure 4).

## 4 DISCUSSION

This is the first human study with intravenous infusion of LKYN, and the major findings were that systemic LKYN administration was safe, tolerable, and had no physiological effects in either species. The increase in LKYN plasma concentration was expected, while lack of change in KYNA and Trp plasma concentrations suggested a relatively slow metabolism of LKYN and no or little feed‐back effect of LKYN on its synthesis. Too fast degradation of KYNA in circulation cannot be excluded. However, preclinical studies showed that the increased KYNA after systemic LKYN administration remained high for 4 hours compared with baseline despite a slight fall.^32^

Systemic LKYN administration was conducted with different concentrations in different species. In rhesus monkeys, administration of 25 mg/kg LKYN caused a rapid increase in plasma KYNA levels by sixfold.^32^ While the dose used in mice varied between 150 and 300 mg/kg and led to cerebral hypoperfusion without altering mean arterial blood pressure,^33^ the same dose increased the cortical concentration of KYNA and suppressed cortical spreading depression in rats.^34^

To translate animal dose to human equivalent dose (HED), we used the following formula ^35^: $\mathrm{HED}=\mathrm{animal}\mathrm{dose}x\frac{\mathrm{animal}\mathrm{km}}{\mathrm{human}\mathrm{km}}$. Km factor for monkeys is 12, whereas km factor for a 70 kg human is 40. Thus, the dose to be used in humans is 5–10 mg/kg. Since this is the first human study to investigate the physiological effect of intravenous LKYN administration, it was difficult to find the start administration dose. To avoid serious adverse events, we started with a very low dose and performed series of experiments to end up with dose of 5 mg/kg. Higher dose might be needed for the treatment purpose, and their pharmacokinetic properties would be important.

LKYN can be converted to the excitatory amino acid receptor antagonist KYNA by kynurenine aminotransferases (KAT).^36^ Targeting NMDA receptor exhibits an antinociceptive effect in cephalic as well as extra cephalic regions in animal models of pain.^37^, ^38^, ^39^ In terms of drug development, the two individual kynurenines of greatest importance are QUIN and KYNA. QUIN selectively activates NMDA receptors, whereas, KYNA, which is produced from by the kynurenine aminotransferases (KAT), antagonizes them.^1^ Thus, QUIN/KYNA ratio may represent a biomarker for NMDA receptor activity. In the present study, no change was found in KYNA plasma concentration. This observation suggests the following: 1 slow metabolism of LKYN, and thus, blood samples for upcoming studies must be obtained over longer time (>2 hours, 2 higher dose of LKYN must be used to change the systemic KYNA concentration; and 3 difference in KYNA concentration between the cerebrospinal fluid (CSF compared with the plasma, and this is supported by the reported unusual fatigue after the hospital phase.

In both humans and rats, LKYN caused neither vasodilation nor changes in HR and MAP. The doses administered to humans and rats were within the same range but, as the infusion to rats was done via a catheter inserted into the carotid artery (i.c.) the delivery of LKYN to the cranial vasculature, the site of interest in this case, was higher in rat because of less dilution. In a previous study, we found that 7–17‐fold lower i.c. doses were required than i.v. doses to achieve the same effect on intracranial arteries, but without a change in BP.^24^

It has been reported that LKYN is an endothelial‐derived regulator of vascular tone causing an upregulating of cyclic adenosine monophosphate (cAMP) and cyclic guanosine monophosphate (cGMP) concentration, leading to vasodilation.^40^ Interestingly, while the vasoactive factor was initially reported to be LKYN itself,^40^ this effect has recently been attributed to the potential action of xanthurenic acid^41^ or cis‐hydroperoxide ((2S,3aR,8aR)‐3a‐hydroperoxy‐1,2,3,3a, 8,8a‐hexahydropyrrolo [2,3‐b] indole‐2‐carboxylic acid) (cis‐WOOH), an intermediary product of oxidatively activated IDO.^42^ The lack of vascular effect in our study is in line with these findings. Whether higher dose of LKYN might cause vascular changes cannot be ruled out.

Kynurenine metabolites are reduced in chronic migraine patients and cluster headache patients compared with healthy controls.^43^, ^44^ Given that L‐kynurenine may exert vasodilating effects similar to nitric oxide by increasing cyclic guanosine monophosphate and initiating downstream cascades (known to trigger migraine attacks)^40^, ^45^, ^46^, ^47^, it has been suggested that the KP may represent a potential therapeutic target in migraine.^13^ In the present study, LKYN infusion caused no headache. Although the first LKYN dose 50 µg/kg provoked mild headache, the following doses did not induce headache, and therefore, placebo effect might explain the provoked headache. Collectively, the present data suggest that LKYN is unlikely involved in initiation of migraine.

## 5 CONCLUSIONS AND FUTURE PERSPECTIVES

Intravenous infusion of LKYN was safe and well tolerated up to dose of 5 mg/kg. Further investigations are warranted with increasing the dose and including other samples such as CSF and more details from blood samples. Given that preclinical studies suggest an important role of LKYN in focal cerebral ischemia^48^ and epileptic seizures,^49^ the therapeutic potential of LKYN in these conditions should be explored in future studies in humans.

## CONFLICT OF INTEREST

MMK has acted as an invited speaker for Novartis and received travel grant from ElectroCore, LLC. MA is a consultant, speaker, or scientific advisor for Allergan, Amgen, Alder, ATI, Eli Lilly, Novartis, and Teva, primary investigator for Alder, Amgen, Allergan, Eli Lilly, Novartis, and Teva trials. MA has no ownership interest and does not own stocks of any pharmaceutical company. MA serves as associate editor of Cephalalgia, associate editor of Headache, co‐editor of the Journal of Headache and Pain. MA is President of the International Headache Society. JMH, DAK, AH, IJO, KU, JB, and LV declare no conflict of interest.

## AUTHOR CONTRIBUTIONS

MMK, JMH, and MA initiated and contributed to human study design; protocol development; participant enrolment; data acquisition, data processing, analysis, statistics, and interpretation; and drafting and revision of the paper. KU, JB, and LV contributed to human study design, protocol development, kynurenine metabolite analysis, statistical analyses, and critical review of the paper. DAK contributed to participant enrolment, data interpretation, and drafting and revision of the paper. AH and IJO initiated and contributed to animal study design; protocol development; participant enrolment; data acquisition, data processing, analysis, statistics, and interpretation; and drafting and revision of the paper.
